# Design and optimization of peptide nanoparticles

**DOI:** 10.1186/s12951-015-0119-z

**Published:** 2015-10-24

**Authors:** Tais A. P. F. Doll, Raja Dey, Peter Burkhard

**Affiliations:** Institute of Materials Science, University of Connecticut, Storrs, CT 06269-3136 USA; Department of Molecular and Cell Biology, University of Connecticut, Storrs, CT 06269-3125 USA

**Keywords:** Self-assembly, Peptide nanoparticles, Drug delivery, Dynamic light scattering

## Abstract

**Background:**

Various supra-molecular structures form by self-assembly of proteins in a symmetric fashion. Examples of such structures are viruses, some bacterial micro-compartments and eukaryotic vaults. Peptide/protein-based nanoparticles are emerging in synthetic biology for a variety of biomedical applications, mainly as drug targeting and delivery systems or as vaccines. Our self-assembling peptide nanoparticles (SAPNs) are formed by a single peptide chain that consists of two helical coiled-coil segments connected by a short linker region. One helix is forming a pentameric coiled coil while the other is forming a trimeric coiled coil.

**Results:**

Here, we were studying in vitro and in silico the effect of the chain length and of point mutations near the linker region between the pentamer and the trimer on the self-assembly of the SAPNs. 60 identical peptide chains co-assemble to form a spherical nanoparticle displaying icosahedral symmetry. We have stepwise reduced the size of the protein chain to a minimal chain length of 36 amino acids. We first used biochemical and biophysical methods on the longer constructs followed by molecular dynamics simulations to study eleven different smaller peptide constructs. We have identified one peptide that shows the most promising mini-nanoparticle model in silico.

**Conclusions:**

An approach of in silico modeling combined with in vitro testing and verification yielded promising peptide designs: at a minimal chain length of only 36 amino acids they were able to self-assemble into proper nanoparticles. This is important since the production cost increases more than linearly with chain length. Also the size of the nanoparticles is significantly smaller than 20 nm, thus reducing the immunogenicity of the particles, which in turn may allow to use the SAPNs as drug delivery systems without the risk of an anaphylactic shock.

**Electronic supplementary material:**

The online version of this article (doi:10.1186/s12951-015-0119-z) contains supplementary material, which is available to authorized users.

## Background

Proteins of varied structures have evolved in nature to self-assemble into spherical nanoparticles. Examples of such supra-molecular structures are viruses [1], some bacterial micro-compartments [2] or eukaryotic vaults [3], which form by self-assembly of folded proteins in a symmetric fashion. They often perform sophisticated cellular functions. Such nanoparticles have a central cavity that can be exploited as a simple encapsulation system for the transport and controlled release and delivery of drugs and genes to targeted cells [4–6]. They can also be used in protein separation, enzyme immobilization, and in blood cell substitution [7, 8]. Virus-like particles have long been used as vaccine platforms for infectious diseases but they could potentially also be used as therapeutic vaccines to treat addiction and other diseases such as cancer [9, 10]. In our group we are designing self-assembling peptide nanoparticles (SAPNs) as vaccines for infectious diseases like malaria [11], HIV [12] or influenza [13], but they can be used for many other diseases [14].

**Fig. 1:**
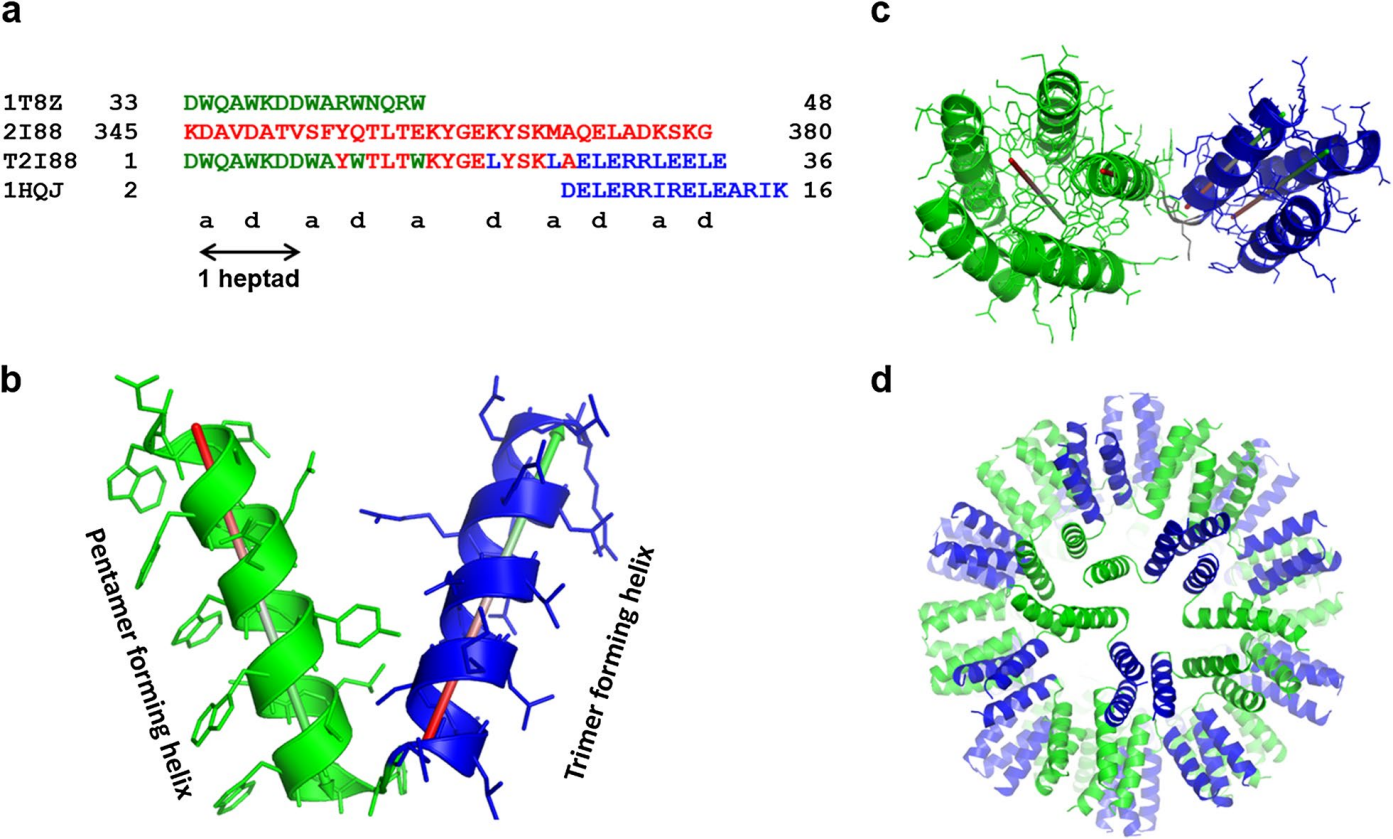
Amino acid sequence and three-dimensional model of peptide building block, pentamer-trimer complex, and mini-nanoparticle. **a** Amino acid sequence from the tryptophan zipper [PDB:1T8Z] and a de novo designed trimeric coiled-coil peptide [PDB:1HQJ] aligned with the sequence of the channel-forming domain of colicin E1 [PDB:2I88]. A parent sequence T2I88 is shown below that has been followed to build the initial model of the mini-nanoparticle peptide. Computational model of the starting construct (peptide 1) (**b**), neighboring pentamer-trimer complex (**c**), and a mini-nanoparticle (**d**) were built using the initial model of the peptide

Our SAPNs are formed by a single peptide chain that consists of two helical coiled-coil segments connected by a short linker region. One helix is forming a pentameric coiled coil while the other is forming a trimeric coiled coil (Fig. 1a, b). Coiled coils are an ubiquitous protein folding and oligomerization motif that exhibits abundance in sequence and function [15]. For instance, coiled coils like bZIP transcription factors contain a DNA-binding sequence whereas other coiled coils like intermediate filaments and spectrin can be structural components of the cell [16]. Furthermore, coiled coils can have dynamic functions such as myosin and dyneins, which act as “movement” proteins [16]. The majority of α-helical coiled coils are based on a heptad sequence repeat, composed of seven amino acids usually denoted **abcdefg** [17]. From two to seven strands of α-helices wrap around each other to create a superhelical twist that is normally left-handed [15]. The driving force for the interaction between these α-helices are the hydrophobic amino acids located in positions **a** and **d** of the heptad repeat. Ionic interactions between residues, for example in position **e** and **g** of the heptad repeat (*i* to *i* + *5*) in parallel dimers and trimers, also further stabilize coiled-coil interfaces.

Coiled coil stability can further be influenced by a number of factors including helical propensity of the residues, helix dipole, helix capping effects, translational and rotational entropy contributions and length of the coiled coil [15]. Litowski et al. investigated the effect of chain length in their heterodimeric coiled-coil system used in applications such as biosensors and affinity chromatography. Their study showed that an increase in chain length led to an increase in stability of heterodimeric coiled coils, however, the effect was nonlinear [18].

In a departure from studies of chain length effects, we have previously aimed to design the shortest possible coiled coil peptide using principles that play a part in monomeric α-helical stability or oligomeric packing. Interestingly, a two heptad-repeat peptide was produced that was 100 % dimeric under physiological buffer conditions [19, 20].

Here we have further investigated the influence of the length of the coiled coil for the self-assembly of our SAPNs, notably the length of the de novo designed trimer. There are many factors that govern the self-assembly of the peptide nanoparticles such as the nature of the linker region, constitution of pentamer and trimer. In a first step we decided to investigate the effect of the trimer length on the self-assembly of the SAPNs. Apart from inherent scientific curiosity, useful applications of this study would be potential chemical syntheses of the monomeric polypeptide instead of production with recombinant protein technology. Chemically synthesizing a long polypeptide is expensive, hence a shorter polypeptide displaying the same properties would be very advantageous and it would also allow for easier chemical functionalization.

**Fig. 2:**
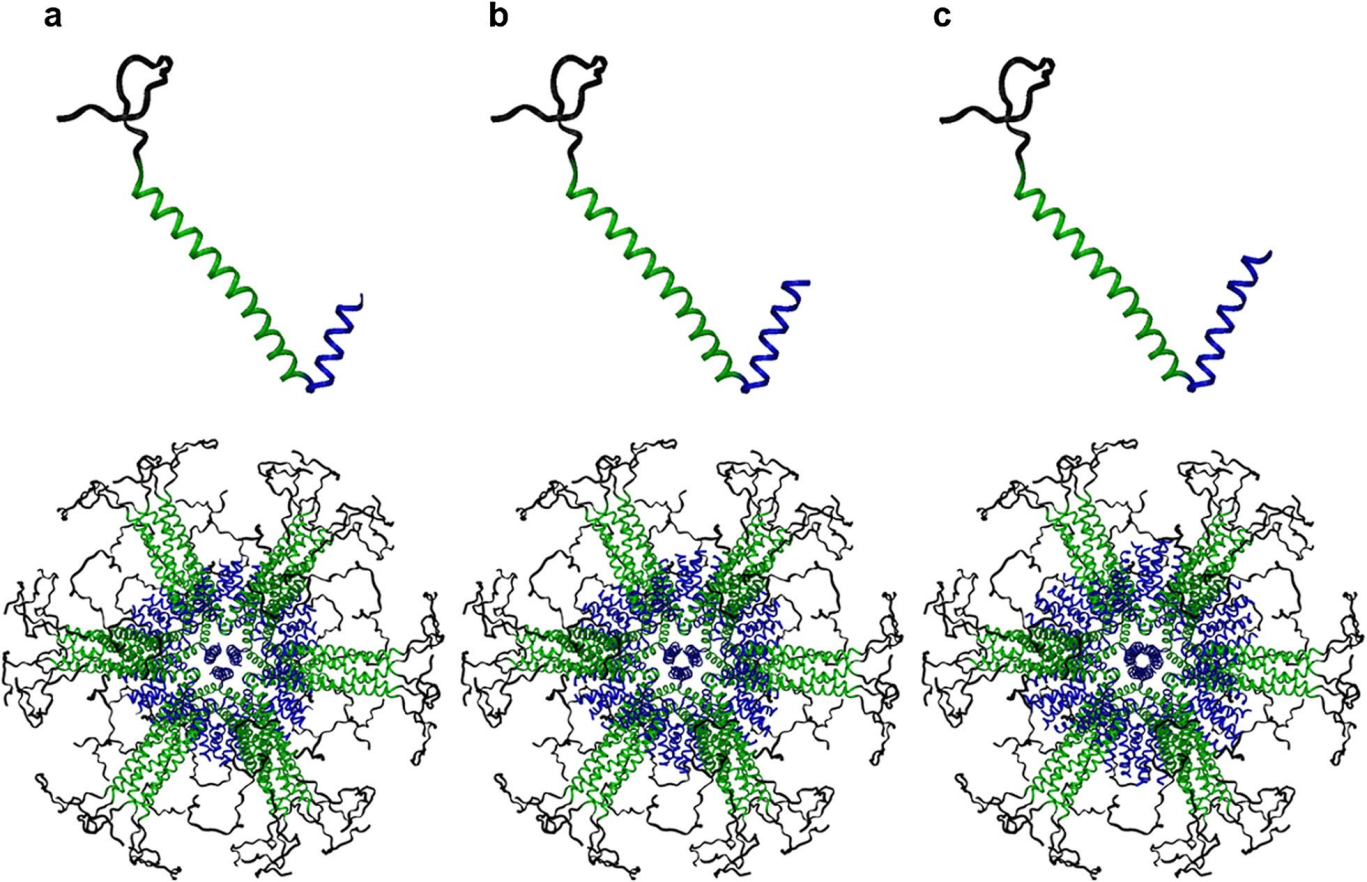
Monomeric building block and computer models of complete SAPNs. Top: monomeric building block composed of a His-tag sequence (*black*), a pentameric domain based on Trp-zipper (*green*) and a shortened trimeric de novo designed coiled-coil domain (*blue*). *Bottom* Computer models of the complete peptide nanoparticle with a diameter of roughly 21 nm. The *black region* (His-tag) is a random coil, which could be fully extended giving a diameter of roughly 27 nm. **a** 2HR, **b** 2.5HR and **c** 3HR

**Fig. 3:**
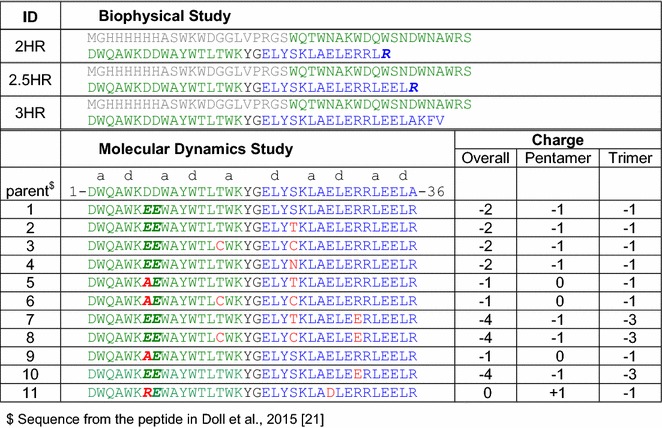
Amino acid sequences of the designed constructs used for biophysical and molecular dynamics simulation study

Three constructs were designed, expressed, purified and refolded under different buffer compositions (Figs. 2 and  3). It was found that even with only 2.5 heptad repeats in the trimer peptide nanoparticles were formed. Furthermore, pH and salt concentration were shown to greatly affect the size and aggregation state of the SAPNs.

Apart from the length of the coiled coils designing and optimizing the correct linker has a pivotal role in the development of a nanoparticle that correctly refolds in vitro. We have done an exhaustive search in the protein data bank to find a template for a helix-turn-helix motif in which the angle between the two helices is about 38°, corresponding to the angle between the fivefold and threefold axes in an icosahedron. Our recent study [21] has shown that such a motif of the crystal structure of colicin E1 [PDB:2I88] gives the optimum template for the linker (Fig. 1a), which can covalently connect two neighboring helices to produce correctly folded nanoparticles. In a second step we try to further optimize this linker region by the use of molecular dynamics simulations of the small peptide.

All these different mutants along with the initial parent peptide (Fig. 3) were studied by molecular dynamics (MD) simulations using the program CHARMM 36b1 in an attempt to further optimize the linker region and find the best peptide building block for the SAPNs.

## Results

### Biophysical analysis

From the linker constitution study it was found that a malaria nanoparticle vaccine construct self-assembled into almost spherical nanoparticles [21]. The de novo trimeric coiled coil in this construct had 6.5 heptad repeats (46 residues). This led us to the question: if the trimeric coiled coil was shortened would nanoparticles still be formed? To answer this question the original construct was genetically engineered by PCR-mediated site-directed mutagenesis to create three new constructs. Their sequences can be found in Fig. 3 and molecular models of the three designs are shown in Fig. 2.

The SDS-PAGE gel for *3HR* showed that this construct expressed well. Elution from the Ni-affinity column took place at pH 4.5 and also in a buffer containing 250 mM imidazole. The predicted molecular weight for *3HR* is 10,251.3 Da.

**Fig. 4:**
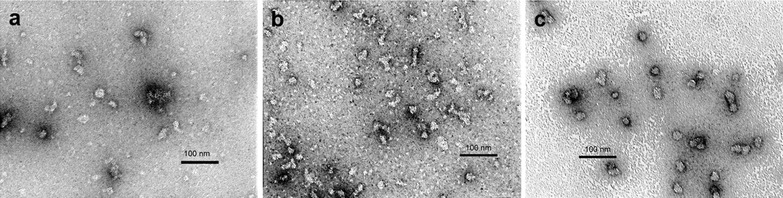
Electron micrograph of SAPNs with different chain length. **a** Electron micrograph of 3HR at pH 10.5, **b** Electron micrograph of 2.5HR at pH 10.5, and **c** Electron micrograph of 2.5HR at pH 9.5

Stepwise refolding at pH 7.0 and pH 8.0 was attempted but resulted in aggregation. Direct refolding at pH 10.5 (20 mM CAPSO, 450 mM NaCl, 5 % glycerol) resulted in nanoparticles as can be observed from TEM and DLS (Fig. 4a, Additional file 1: Figure S1). However, it is evident from both, the DLS and the TEM data, that the preparation is significantly heterogeneous.

The SDS-PAGE gel for *2.5HR* showed a similar pattern to *3HR* except there also seemed to be elution at pH 5.2. The predicted molecular weight for *2.5HR* is 9805.7 Da.

The first refolding scheme attempted for *2.5HR* was direct refolding (dialysis against a buffer with no urea). Direct refolding at pH 10.5 (20 mM CAPSO, 50 mM NaCl, 5 % glycerol) resulted in nanoparticles as can be observed from TEM and DLS but also aggregation (Fig. 4b; Additional file 1: Figure S1).

To further characterize *2.5HR* a pH screening was performed. Briefly, the quick refolding method, where the protein is concentrated to 1 mg/ml then diluted 20 times to a buffer with no urea, was used. For the buffers used for dilution the salt concentration (50 mM NaCl) and glycerol volumes (5 %) were kept constant, only varying the buffering agent for each pH (pH 7.5–20 mM Hepes, pH 8.5–20 mM Tris, pH 9.5–20 mM CAPSO). At pH 7.5 precipitation in the dialysis bag was observed. At pH 8.5 there seemed to be no aggregation visible to the naked eye. However, investigation by TEM revealed clusters of aggregation. Only at pH 9.5 were there very nice nanoparticles visible (Fig. 4c).

We decided to further screen different salt concentrations at this pH to confirm nanoparticle assembly. The quick refolding method described above was used. The different buffers had the same pH 9.5 (20 mM CAPSO) and same glycerol concentration (5 %) only the salt concentration varied: no salt, 50 mM NaCl, 100 mM NaCl and 400 mM NaCl. It seems that as the salt concentration is increased the nanoparticle diameter also increases (Additional file 2: Figure S2).

The construct with the shortest trimer was *2HR*. Elution from the Ni-affinity column occurred largely at pH 5.2 but also at pH 6.3 and at pH 5.9. However, direct refolding at pH 10.5 (20 mM CAPSO, 50 mM NaCl, 5 % glycerol) resulted in aggregation. Refolding of 2HR under pHs 9.5 and 8.5 also resulted in aggregation. Since this aggregation was visible to the naked eye it wasn’t evaluated by DLS or TEM so results were not included.

SAPN was settled less than 24 h for DLS measurement and the suspension was not diluted. For accuracy purposes five scans were collected. The goal of this study was to characterize SAPNs immediately after refolding and long term stability was not evaluated. Previous research efforts in our group shows that both long term settling of SAPNs and their concentration might or might not affect the morphology of SAPNs. It depends upon protein sequence, storage temperature and buffer [22].

### Molecular dynamics simulation

**Fig. 5:**
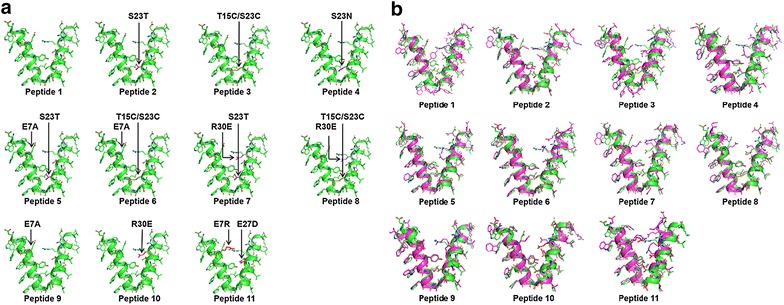
Initial models and their deviations from corresponding final models after 2 ns of molecular dynamics simulation. **a** Initial models of 11 constructs, where peptides 2 to 11 have been built by in silico mutation on the starting model (peptide 1). Mutations have been indicated by arrows. **b** Nanoparticle peptides of the 11 constructs (shown in *magenta color*) after 2 ns of molecular dynamics simulation superposed on their corresponding initial peptides (shown in *green color*)

Based on our construct design and the biophysical results of the *2.5HR* we ran a series of molecular dynamics simulations on the eleven short versions of *2.5HR* including only five helical turns (i.e. 2.5 heptad repeats) around the linker region of the nanoparticle peptide (Figs. 3, 5a) in an attempt to further optimize the sequence that would yield the best refolding behavior. The angle between the pentameric and trimeric helices, the overall charge, the charge distribution along the peptide chain, and the different non-covalent interactions within the peptide nanoparticle, along with the solvent condition, are the key parameters that govern the quality of the nanoparticle formed in solution. Figure 3 represents different mutations of the parent sequence of the model peptide that we have studied by molecular dynamics simulation. Mutations have been introduced close to the glycine residue of the linker region of all the constructs. A disulfide bond has been introduced by double mutations T15C and S23C in peptide 3, peptide 6, and peptide 8 to see how this constraint affects the peptide conformation. A single mutation S23T on the trimeric side has been introduced in peptide 2, peptide 5, and peptide 7 to see how this bulkier residue (threonine versus serine) can affect the overall conformation of these peptides. Another single mutation S23N on the trimeric side has been tested in peptide 4. Additional mutations have been introduced a little farther away from the linker region to modify the long range electrostatic interactions between the oligomerization domains along the fivefold and threefold axes of the icosahedron. Figure 5a represents the 3D models of all the initial peptides 1 to 11 with the mutant residues in red color. The overall charge in both the pentameric and trimeric domains along with the overall charge of the construct are depicted in Fig. 3.

**Fig. 6:**
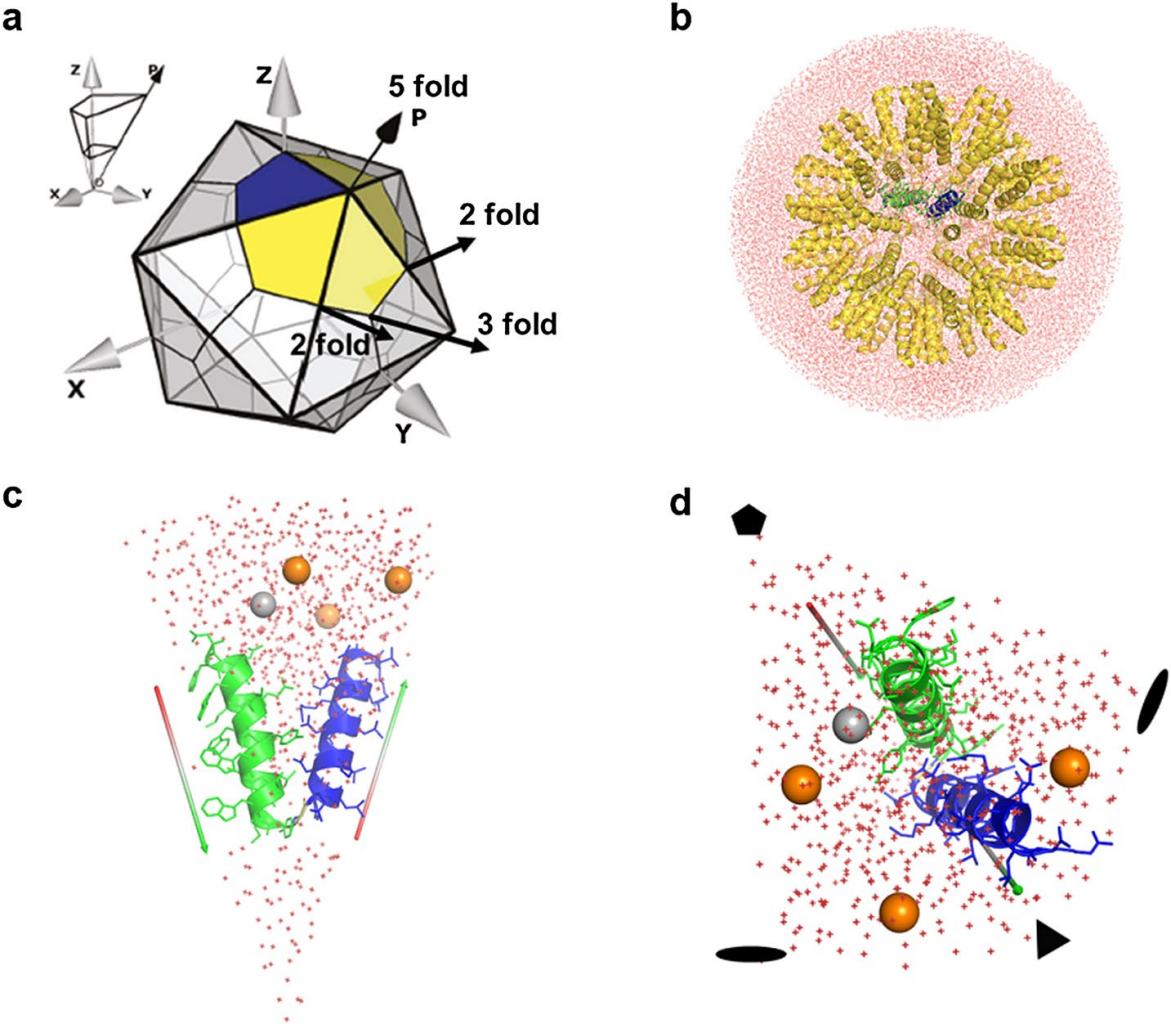
Building asymmetric unit for molecular dynamics simulation of nanoparticle. **a** An icosahedron showing twofold, threefold and fivefold axes. **b** A fully solvated nanoparticle in presence of salt ions. Side view (**c**), and top view (**d**) of the asymmetric unit of an entirely solvated nanoparticle in presence of salt ions

Simulating a complete nanoparticle with spherical water around it is very time-consuming as the whole solvated system contains about half a million variables (Fig. 6b). In contrast, using only an asymmetric unit of the icosahedron during molecular dynamics runs and simulating the rest of the particle by the icosahedral symmetry operations applied to the asymmetric unit, the system becomes computationally much more efficient. An asymmetric unit can be built by cutting the whole system along the four planes passing through the rotational axes fivefold and twofold, twofold and threefold, threefold and twofold, and twofold and fivefold (Fig. 6a). The side and top views of such a wedge-shaped asymmetric unit containing only one nanoparticle peptide, water, and ions are shown in Fig. 6c, d. During MD simulations only the interactions within this wedge and its symmetry-related neighbors are calculated, which makes the MD runs feasible.

**Fig. 7:**
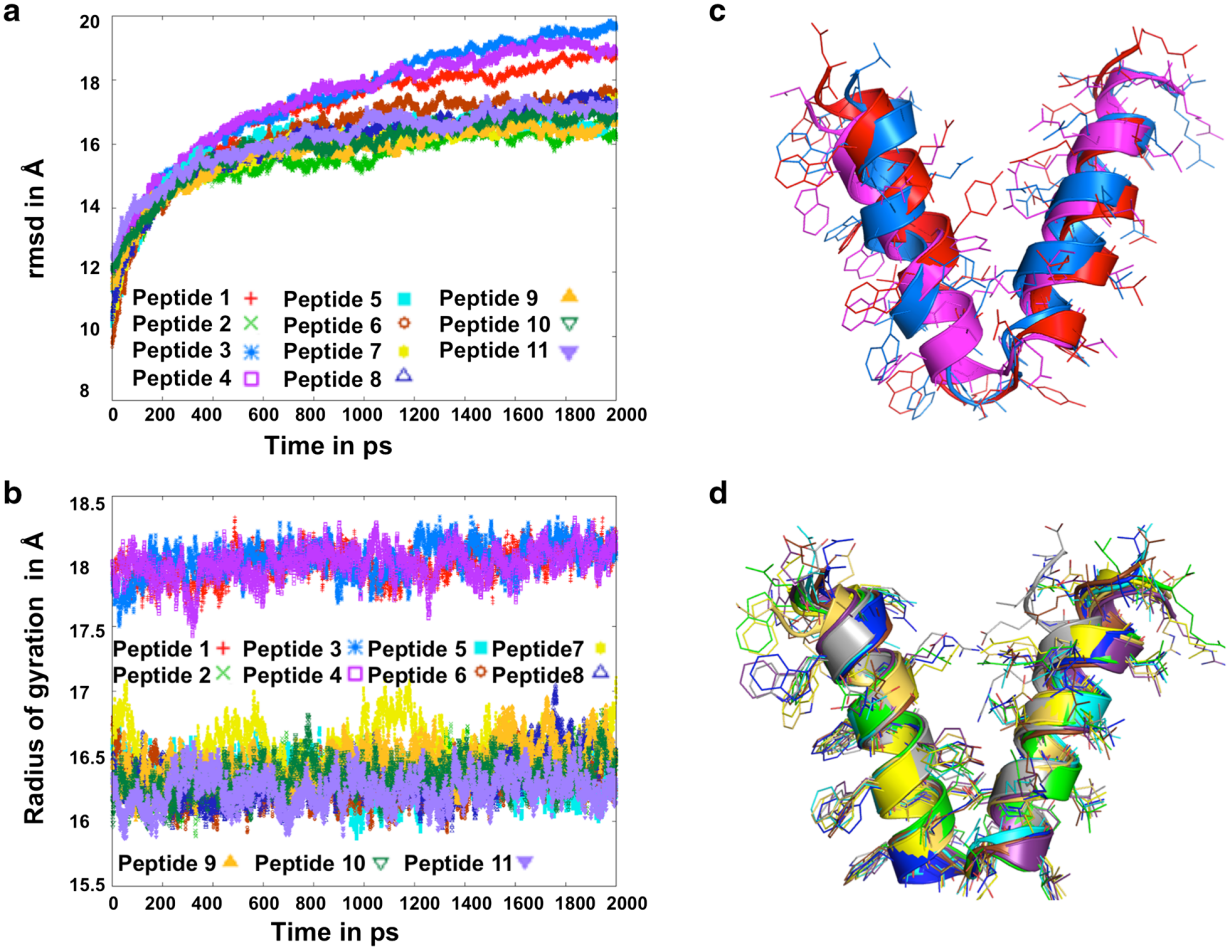
Molecular dynamics simulation of 11 peptides for 2 ns. RMS deviations (**a**) and radius of gyrations (**b**) of 11 peptides relative to their corresponding energy minimized structures after 2 ns of MD simulation. **c** Superposition of 3 diverging peptides 1, 3, and 4. **d** Superposition of 8 converging peptides 2, 5, 6, 7, 8, 9, 10, and 11

The resulting peptide structures after a 2 ns MD simulation have been superposed on their corresponding initial models (Fig. 5b). Variations of RMSD with time of simulation have been calculated in 100 mM sodium chloride solution (Fig. 7a) for the eleven models chosen initially. Another structural characteristics is the radius of gyration (RGYR), which is a combined measure of its overall size and shape.

Here, we have calculated the RGYR in 100 mM salt environment, which are plotted in Fig. 7b. Panels a and b show two clusters of molecules in the salt environment, one with a diverging tendency (peptides 1, 3, and 4) and the other eight constructs (peptides 2, 5, 6, 7, 8, 9, 10, and 11) with a converging tendency. Eight converging peptides show the variation of RMSD within 7 Å with respect to the energy minimized structure, whereas the variation of RMSD for the three diverging structures is about 10 Å. Again, Fig. 7b shows that the RGYR are pretty stable with simulation time, and the value (16.2 Å) for the converging cluster is lower than that of the diverging cluster (18 Å). MD simulations have also been run in water environment (data not shown) showing a tendency of divergence indicating unstable conformation even after 2 ns. Thus, adding salt has an important role in folding and converging to a stable nanoparticle. Figure 7c shows the superposition of 3 diverging constructs (peptides 1, 3, and 4) after 2 ns of MD simulation. Peptides 1 and 3 show deformation at the linker region, while peptide 4 shows deformation at the N-terminus of the pentameric helix. In both models, where the linker region contains either a disulfide bond between two cysteine residues (peptide 3) or a hydrogen bond between threonine and asparagine (peptide 4), a deformation of the helices can be observed. Similarly, an unbalanced situation in the start model (peptide 1) results in a structural deformation as well (Fig. 7c). The upper clusters in Fig. 7a, b that shows the divergence behavior of three model peptides (peptides 1, 3, and 4) during 2 ns molecular dynamics simulation in presence of 100 mM NaCl, were discarded for further analysis. Figure 7d shows the superposition of the remaining eight constructs (peptides 2, 5, 6, 7, 8, 9, 10, and 11), which are converging during 2 ns MD simulation in presence of 100 mM NaCl as shown in the lower clusters of the Fig. 7a, b.

Additional file 3: Figure S3 shows the effect of additional mutation on peptide 2, while Additional file 4: Figure S4 shows the effect of an additional mutation on peptide 3. In the model of peptide 2 (with an overall charge of −2), where the serine residue in the trimeric domain was replaced by threonine (S23T), no deformation of the helices was observed during a 2 ns MD simulation. Rather, it converged into a stable helical conformation. Although peptide 2 converged to a reasonably good structure at the end of MD simulation, when supplemented with an additional mutation (E7A or R30E) (peptides 5 and 7), it showed some degree of deformation in the 3D structure and also a deviation in rmsd and radii of gyration (Additional file 3: Figure S3). Specifically, in peptide 7, where the mutation R30E causes a loss of salt bridge interaction between E7 and R30 in peptide 2, a deformation developed at the N-terminus of the pentameric helix (Additional file 3: Figure S3d). Although peptide 2 and peptide 5 did not show much structural deformation during 2 ns MD simulation, the angles between the pentamer and trimer helices are 49.9° and 45.7° respectively, which deviate by 12.5° and 8.3° from the angle (37.4°) between the corresponding fivefold and threefold axes in an icosahedron (Fig. 4a). The angle between the two helices in peptide 7 is 34.3°, which is 3.1° smaller than the value in the icosahedron. Final models of peptides 9 and 10 after 2 ns MD simulation are shown in Fig. 5. Alanine substitution in place of Glu7 causes a deformation at the N-terminus of the pentameric helix in peptide 9. However, the reduced electrostatic interaction caused by the R30E mutation in the trimeric helix of peptide 10 results in maintaining the overall conformation with a slight bending at the C-terminus of this helix (Fig. 5b). The double mutation E7R/E27D introduced in peptide 11 (Fig. 5b) causes a deformation in the trimeric helix while maintaining the helical conformation of the pentameric domain. The mutation E7R on the pentameric domain probably repels R30 on the trimeric side, thereby initiating both intra- and inter-domain salt bridge interactions R30 to D27 and R7 to E34 respectively that lead to a distinct conformational change at the C-terminus of the trimeric helix in peptide 11.

**Fig. 8:**
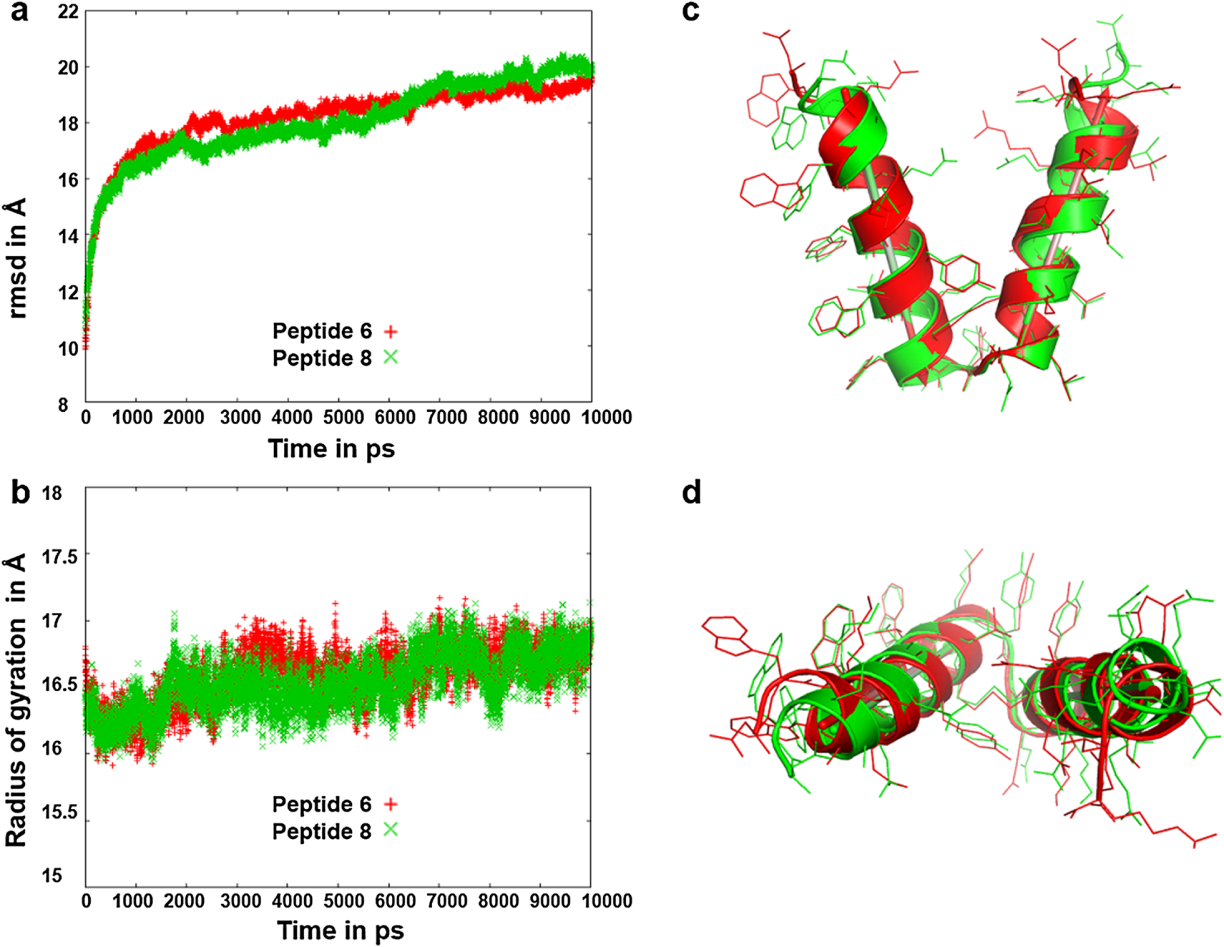
Molecular dynamics simulation of two peptides 6 and 8 for 10 ns. RMS deviations (**a**) and radius of gyrations (**b**) of the two peptides 6 and 8 relative to their corresponding energy minimized structures after 10 ns of MD simulation. Side view (**c**) and top view (**d**) of the superposition of the molecular structure of peptide 6 (in *red color*) on peptide 8 (in *green color*) after 10 ns of MD simulation

By carefully analyzing the 8 converging models in 3D, we were able to identify the two peptide models (peptide 6 and 8) showing lower rmsd and radii of gyrations with a reasonable angle between the trimer and pentamer while maintaining helical conformations as their secondary structure. Finally, these two models, peptides 6 and 8, were selected based on the nature of convergence (Additional file 4: Figure 5a, b) and in silico visualization on which we further performed MD simulations for a longer period of 10 ns (Fig. 8). Interestingly, the peptide 3 model that shows deformation in MD simulation because of the disulfide bond at the linker region, adopts an adequate helical conformation when supplemented with the same additional mutation E7A or R30E (peptides 6 and 8) beyond the linker region as that studied in peptides 5 and 7 respectively.

The best model has been selected based on a 10 ns MD simulation on these two peptides 6 and 8 (Fig. 8). In Fig. 8a, peptide 8 shows a slightly lower RMSD than that observed in peptide 6 during the first 6.5 ns simulation. Slightly lower radius of gyration for peptide 8 (Fig. 8b) also indicating more compactness than peptide 6. The angle between the pentameric helix and the trimeric helix of our spherical nanoparticle can be greater than the theoretical value 37.4° (calculated between the fivefold and the threefold axes in an icosahedron) depending on the flexibility of the linker region. Here, in peptides 6 and 8 these inter-helical angles are 42.5° and 38.8°. While, the minimum value 37.4° corresponds to the perfect alignment of pentameric and trimeric helices along the fivefold and threefold axes of the icosahedron respectively, the coiled coil add additional twisting to those helical axes. Analyzing all these observations, we concluded that peptide 8 is much closer to an asymmetric unit of an ideal T1 icosahedron and considered to be the best model out of all 11 constructs.

## Discussion

The purpose of this study was to investigate the effect of trimer length on the self-assembly of peptide nanoparticles. Given that original malaria construct [21] formed nice nanoparticles this construct was selected to have its trimer shortened and the epitope removed. The strategy used was to engineer a stop codon by PCR-mediated site-directed mutagenesis at specific locations in the trimer resulting in three different lengths (3, 2.5 and 2) of the heptad repeats in the trimer. Just before the stop codon an arginine residue was introduced so as to further stabilize the short trimeric coiled coil. The hypothesis was that the arginine’s positive charge would counteract the negative charge of the carboxy terminus and make the coiled-coil interaction as strong as possible.

It is clear from the results with all three designs the crucial role pH plays in the self-assembly of the SAPNs. The monomeric peptide consists of a fusion protein where the pentameric coiled coil is connected by a short linker to the trimeric coiled coil. Because of icosahedral symmetry the pentamer and trimer are at an angle where residues close to the linker region from the pentamer can interact with residues from the trimer. If the overall charge in the pentamer is the same as the overall charge in the trimer then they will repel each other. On the other hand, if the pentamer and trimer have opposite overall charges then they will attract each other (see Fig. 3).

Another interesting effect is salt concentration on nanoparticle size (Additional file 2: Figure S2). For *2.5HR* it seems that as the concentration of NaCl is increased, larger nanoparticles are obtained.

Given that the trimer length for SAPNs to self-assemble are 3 and 2.5 heptad repeats, the next logical step was to investigate if self-assembly occurs with a shorter pentamer. How short can you make the pentamer and trimer and still get nanoparticle assembly? To assess the feasibility of future lab work on such minimized peptides, we have performed molecular dynamics studies. Our observation on peptide-based mininanoparticles based on molecular dynamics simulations elucidated the dependencies of point mutations close to and nearby the linker region on the assembly of simulated nanoparticles. Flexibility in the linker region induced by a G19 can be controlled by the design and optimization of a single/double mutation near the linker region. We also studied the effect on the nanoparticle assembly of some point mutations further away from the linker region. A molecular dynamics simulation of 11 designed constructs showed the variation of convergence into helical conformation of the two oligomerization domains as well as that of the inter-helical angle. Peptide 8 folded into the best-assembled nanoparticle after 10 ns molecular dynamics simulation. In this construct we incorporated a disulfide bond by double mutations T15C and S23C together with a third point mutation A30E in the trimeric domain. Although the disulfide bond alone (peptide 3) distorted the peptide conformation, when the additional mutation R30E was included (peptide 8), it converged into a well-folded nanoparticle.

This computational study on peptide mini-SAPNs could potentially benefit the nanotechnology of therapeutic development, especially in vaccine design, drug delivery, and bio-imaging. If you could get a so-called “mini nanoparticle” chemical peptide syntheses might be useful to manufacture the peptide instead of relying on recombinant production with *E. coli.* For vaccine research chemical syntheses of peptide nanoparticles would be advantageous, as traditional contaminants inherent to protein purification such as LPS and proteases would be eliminated. This in turn would allow the peptide nanoparticle to be on “fast-track” to Phase I clinical trials and major hurdles with the FDA could be avoided. For example Audran et al. have led a synthetic peptide derived from a Malaria antigen into a phase I Malaria vaccine trial [23].

## Conclusions

Our investigation demonstrated that the protein conformation is dependent on point mutations within and nearby the linker region to allow proper self-assembly of the SAPNs. Flexibility in the linker region induced by glycine residues is needed to allow for the helix-turn-helix motif, but on the other hand the protein chain can be stabilized in this conformation by the choice of optimal neighboring residues. We studied the effect of point mutations within and somewhat more distant from the linker region on the nanoparticle assembly. A molecular dynamics simulation of 11 designed peptide constructs showed the variation of convergence into a helical conformation of the two oligomerization domains and a stabilization of the angle between the two coiled-coil domains. Peptide 8 folded into the best-assembled nanoparticle after a 10 ns molecular dynamics simulation. Engineering a disulfide bond into the linker region to stabilize the relative orientation of the pentamer and the trimer distorted the peptide conformation. But when combining a third mutation with the disulfide bond it converged into a well-folded nanoparticle. In conclusion, the particular amino acid sequences of the linker region has a significant effect on the assembly properties of the SAPNs. Combining mutations within the linker itself with mutations more distant from the linker can provide a powerful way to design and optimize its peptide sequence. This computational and biophysical study may allow the engineering of devices that can be used for drug targeting, drug delivery, and bio-imaging.

## Methods

### Cloning

In order to introduce an arginine followed by a stop codon in T2i88-8-pf [21] site-directed mutagenesis PCR was used. The resulting plasmids were transformed into *Escherichia Coli* strain DH5α by heat shock. The resulting ampicilin-resistant colonies were screened for the recombinant gene by sequencing after miniprep (Promega, Madison, WI, USA). Plasmids harboring the desired genes were transformed into the *Escherichia Coli* strain BL21(DE3) expression cells (Novagen, Gibbstown, NJ, USA) and a 20 % glycerol stock of each construct (*3HR*, *2.5HR* and *2HR*) was stored at −80 °C.

### Protein expression

An overnight culture (50 ml) of BL21(DE3) (Novagen, Gibbstown, NJ, USA) cells containing the expression construct was added to LB (3L) supplemented with ampicillin (Fisher, Pittsburgh, PA, USA, 6 ml, 100 mg/ml). The culture was incubated (190 rpm, 37 °C) until the OD600 reached ~0.5–0.6, at which time protein expression was induced (IPTG, Fisher, Pittsburgh, PA, USA, 3 ml, 1 M) and the culture incubated further (190 rpm, 37 °C, 3 h). The cells were isolated by centrifugation (4000*g*, 12 min), and the pellet was stored at −80 °C.

### Protein purification

Purification was done under denaturing conditions (9 M urea). Cell pellet was thawed on ice, resuspended in lysis buffer A which is composed of 9 M Urea, 100 mM NaH_2_PO_4_, 10 mM Tris and 10 mM β-mercaptoethanol, pH 8.0 and lysed by sonication (SONICATOR → 3000 Ultrasonic Liquid Processor, cycle of 4 s pulse at 55 % amplitude followed by a 6 s rest repeated for a 10 min period). The insoluble cell debris was cleared by centrifugation (45 min at 30,500*g*). The supernatant was then incubated with nickel beads (Qiagen, Valencia, CA, USA) for one hour. Possible DNA contamination was removed by a wash with buffer B at pH 8.0: 9 M Urea, 500 mM NaH_2_PO_4_, 10 mM Tris, 10 mM imidazole. Protein contaminants were washed from the column using a pH gradient and 10 mM imidazole in the buffers. The first wash was done with lysis buffer A and the second and third washes at pHs 6.3 and 5.9 respectively with a buffer containing 9 M Urea, 100 mM NaH_2_PO_4_, 20 mM sodium citrate, 10 mM imidazole and 10 mM β- mercaptoethanol. Elution was done at pH 5.2 and pH 4.5 again with 9 M Urea, 100 mM NaH_2_PO_4_, 20 mM sodium citrate, 10 mM imidazole and 10 mM β-mercaptoethanol. Any protein that did not elute at the lower pH washes was eluted with buffers containing high concentration of imidazole (buffer C: 9 M Urea, 100 mM NaH_2_PO_4_, 10 mM Tris, 250 mM imidazole, pH 8.0 or buffer D: 9 M Urea, 100 mM NaH_2_PO_4_, 10 mM Tris, 500 mM imidazole, pH 8.0).

Protein purity was verified by sodium dodecyl sulfate polyacrylamide gel electrophoresis (15 % gel). Following purification, the denatured monomeric peptides were refolded using one of the following three methods: (1) dialysis performed in a stepwise manner (stepwise refolding) (2) one step dialysis against a buffer with no urea (direct refolding) or (3) concentrated (Amicon Ultra centrifugal filter devices, Millipore, Billerica, MA, USA) and diluted to a buffer with no urea (quick refolding). The proteins were filtered with a 0.1 μm polyvinylidene fluoride membrane filter before and after dialysis (Millipore, Billerica, MA, USA, #SLVV 033 RS).

For all three designs (*3HR*, *2.5HR* and *2HR*) the protein concentration was calculated using the absorbance at 280 nm with the extinction coefficient (M^−1^ cm^−1^) and molecular weight (Daltons) of the protein. The extinction coefficient and molecular weight were obtained with ExPASy’s ProtParam tool (http://ca.expasy.org/tools/protparam.html).

### Dynamic light scattering

The hydrodynamic diameter was obtained with a Malvern Zetasizer Nano S equipped with a 633 nm laser using a 3 mm path length quartz suprasil cell. The measurements were done at 25 °C and for each protein five scans were collected.

### Transmission electron microscopy

Transmission Electron Microscopy samples were negatively stained with 1 % uranyl acetate (SPI) at a peptide concentration of 50 μg/ml. Electron micrographs were taken with a Philips EM 300 transmission electron microscope at an accelerating voltage of 80 kV. The micrographs were scanned at 600 dpi.

### Model building

A starting model of the nanoparticle peptide was built using the crystal structures of the helix-turn-helix motif of the channel-forming domain of colicin E1 [24], the tryptophan zipper [25] and a de novo designed trimeric coiled-coil peptide [26] as templates for the linker, the N-terminal pentameric domain and the C-terminal trimeric domain, respectively (Fig. 1a). The model building (Fig. 1b) was done in silico using the graphics program ‘O’ version 12.0.1 [27].

### Molecular dynamics simulation

Eleven different constructs were studied by molecular dynamics (MD) simulations with CHARMM 36b1 [28] installed on a Linux cluster at the Biotech Center of the University of Connecticut. The whole MD simulation procedure is divided into five steps: vacuum minimization, solvation, energy minimization, heating and equilibration, and production dynamics. The peptide molecule was solvated and ions were added to achieve a particular salt concentration. In the energy minimization step, we used the steepest descent (SD) method for 50 steps followed by the adopted basis Newton–Raphson (ABNR) method for 50,000 steps, where each step is 1 fs. After the energy minimization of the whole system, the temperature was raised slowly to 300 K from an initial temperature of 100 K at a rate of 10°/1000 steps to relax the molecule and then to run the equilibration for a total time of 100 ps, including the time to raise the temperature. Production dynamics for 2 ns were run thereafter on the whole system for initial screening. Long 10 ns production dynamics were also performed on selected models to find the best one. To analyze the final models, we have calculated root mean square deviation (RMSD) and radius of gyration (RGYR) with respect to the energy minimized structure using CHARMM algorithm [28].

## Additional files

Additional file 1. Figure S1. Dynamic light scattering experiment of SAPNs with different monomeric chain length. (a) Dynamic light scattering of 3HR at pH 10.5. The DLS graph of 3HR shows some heterogeneity composed of nanoparticles (average diameter = 23.3 nm) and some aggregation. (b) Dynamic light scattering of 2.5HR at pH 9.5. The DLS graph of 2.5HR shows a single peak (average diameter = 23.4 nm) and no aggregation.
Additional file 2. Figure S2. Transmission electron micrographs of 2.5HR at pH 9.5 (20 mM CAPSO) and different NaCl concentrations. (a) 0 mM, (b) 50 mM, (c) 100 mM and (d) 400 mM.
Additional file 3. Figure S3. Molecular dynamics simulation of three peptides 2, 5, and 7 for 2 ns. RMS deviations (a) and radius of gyrations (b) of the three peptides 2, 5, and 7 relative to their corresponding energy minimized structures after 2 ns of MD simulation. (c) Superposition of the molecular structure of peptide 5 on peptide 2 after 2 ns of MD simulation. (d) Superposition of the molecular structure of peptide 7 on peptide 2 after 2 ns of MD simulation.
Additional file 4. Figure S4. Molecular dynamics simulation of three peptides 3, 6, and 8 for 2 ns. RMS deviations (a) and radius of gyrations (b) of the three peptides 3, 6, and 8 relative to their corresponding energy minimized structures after 2 ns of MD simulation. (c) Superposition of the molecular structure of peptide 6 on peptide 3 after 2 ns of MD simulation. (d) Superposition of the molecular structure of peptide 8 on peptide 3 after 2 ns of MD simulation.
