# Supplementary figures and images for: Design and optimization of peptide nanoparticles
Source: J Nanobiotechnology. 2015 Oct 24;13:73. doi: 10.1186/s12951-015-0119-z (PMC4619341; doi:10.1186/s12951-015-0119-z)

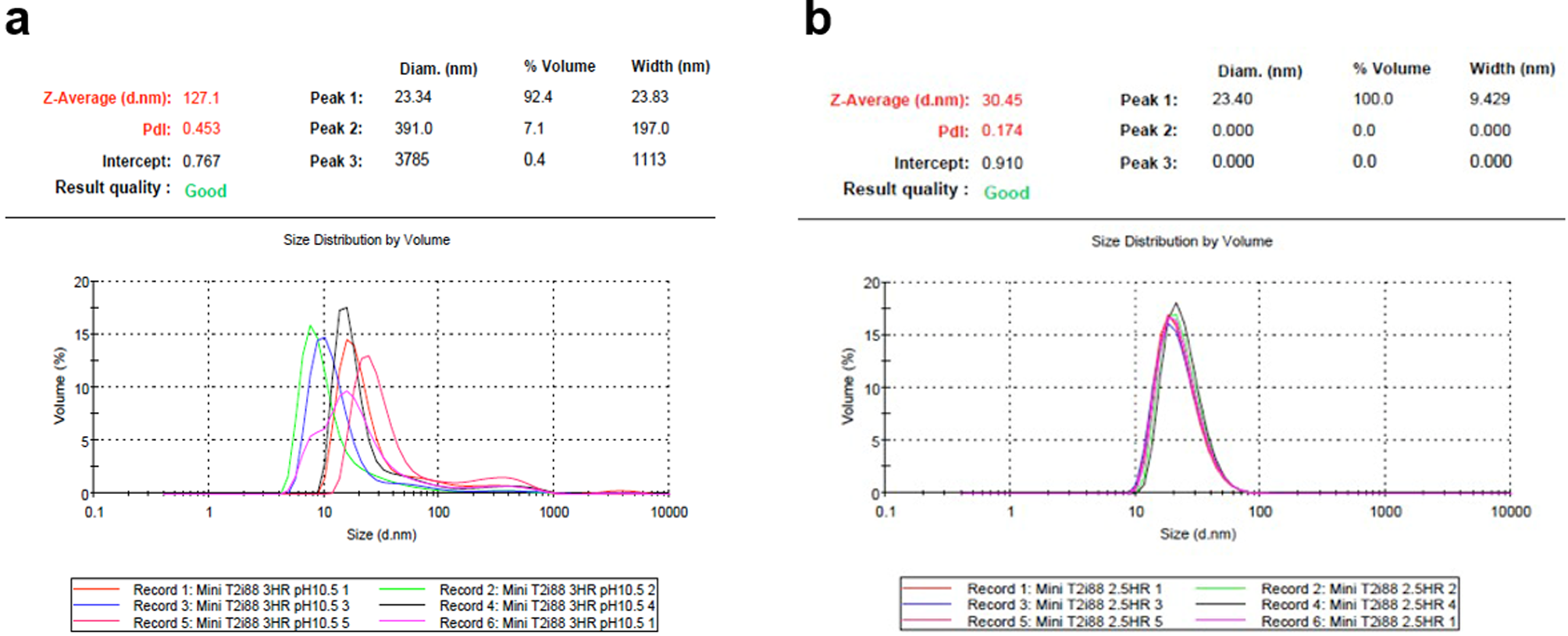

Supplement: Supplementary file 1 — Additional file 1. Figure S1. Dynamic light scattering experiment of SAPNs with different monomeric chain length. (a) Dynamic light scattering of 3HR at pH 10.5. The DLS graph of 3HR shows some heterogeneity composed of nanoparticles (average diameter = 23.3 nm) and some aggregation. (b) Dynamic light scattering of 2.5HR at pH 9.5. The DLS graph of 2.5HR shows a single peak (average diameter = 23.4 nm) and no aggregation. [file 12951_2015_119_MOESM1_ESM.tif]

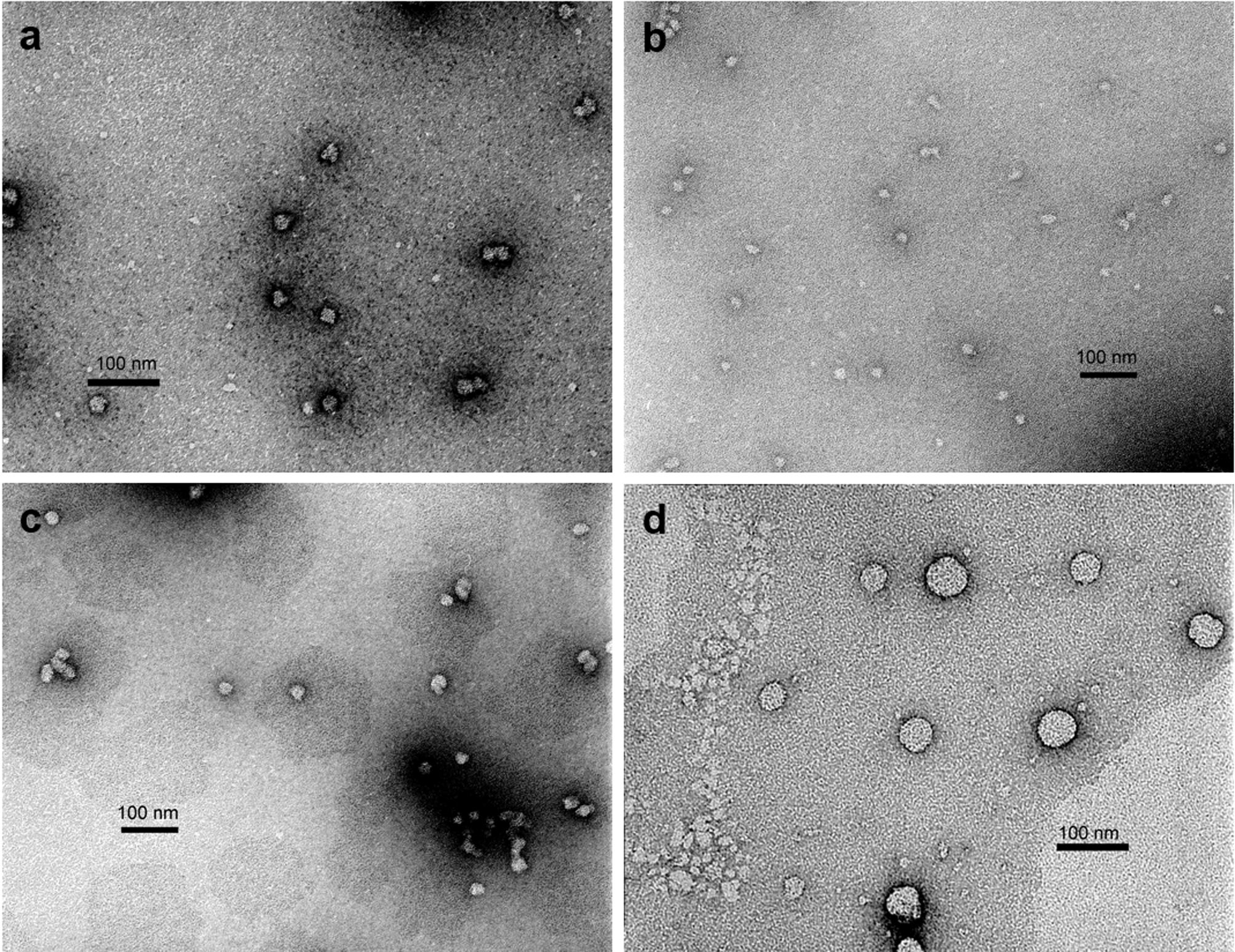

Supplement: Supplementary file 2 — Additional file 2. Figure S2. Transmission electron micrographs of 2.5HR at pH 9.5 (20 mM CAPSO) and different NaCl concentrations. (a) 0 mM, (b) 50 mM, (c) 100 mM and (d) 400 mM. [file 12951_2015_119_MOESM2_ESM.tif]

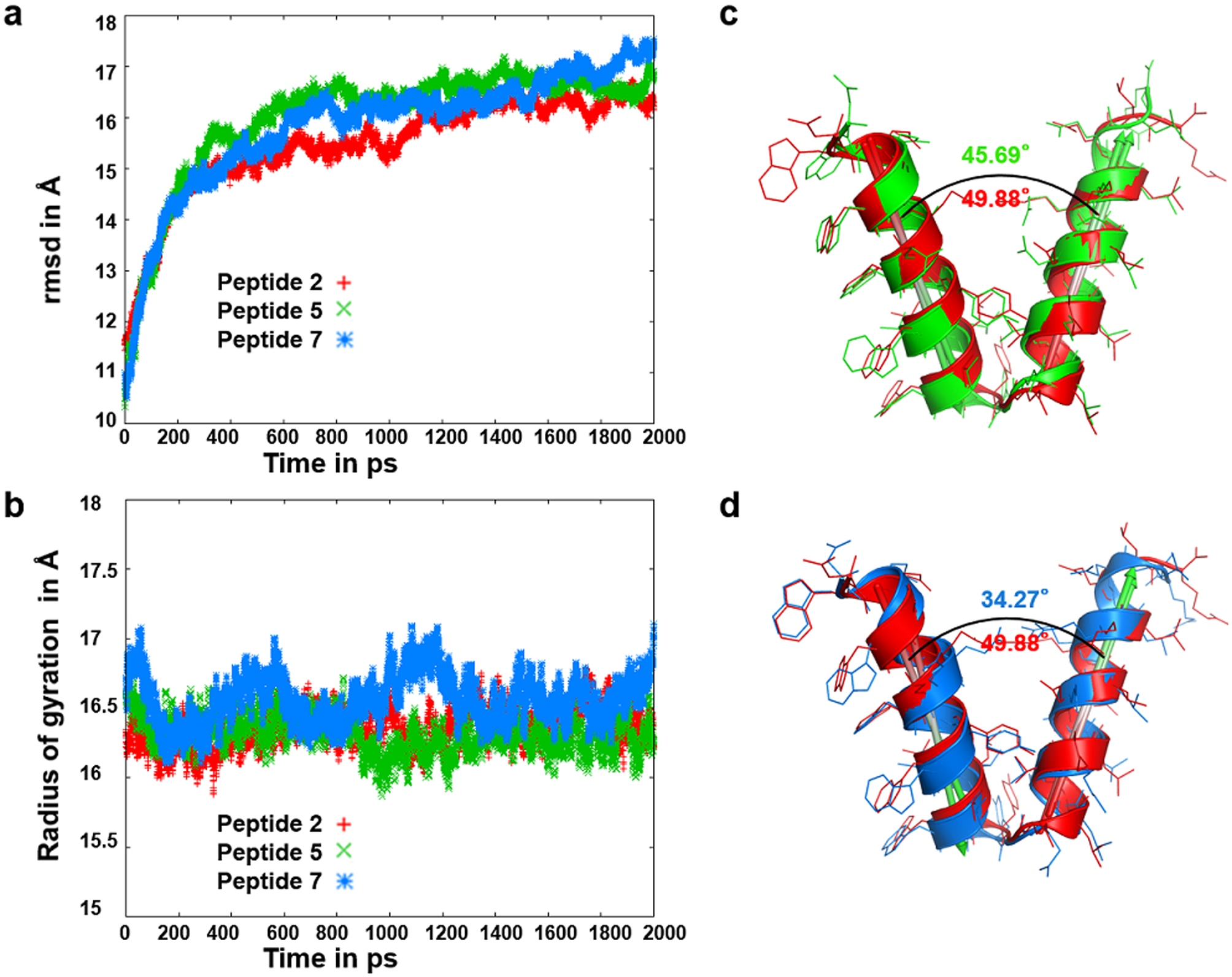

Supplement: Supplementary file 3 — Additional file 3. Figure S3. Molecular dynamics simulation of three peptides 2, 5, and 7 for 2 ns. RMS deviations (a) and radius of gyrations (b) of the three peptides 2, 5, and 7 relative to their corresponding energy minimized structures after 2 ns of MD simulation. (c) Superposition of the molecular structure of peptide 5 on peptide 2 after 2 ns of MD simulation. (d) Superposition of the molecular structure of peptide 7 on peptide 2 after 2 ns of MD simulation. [file 12951_2015_119_MOESM3_ESM.tif]

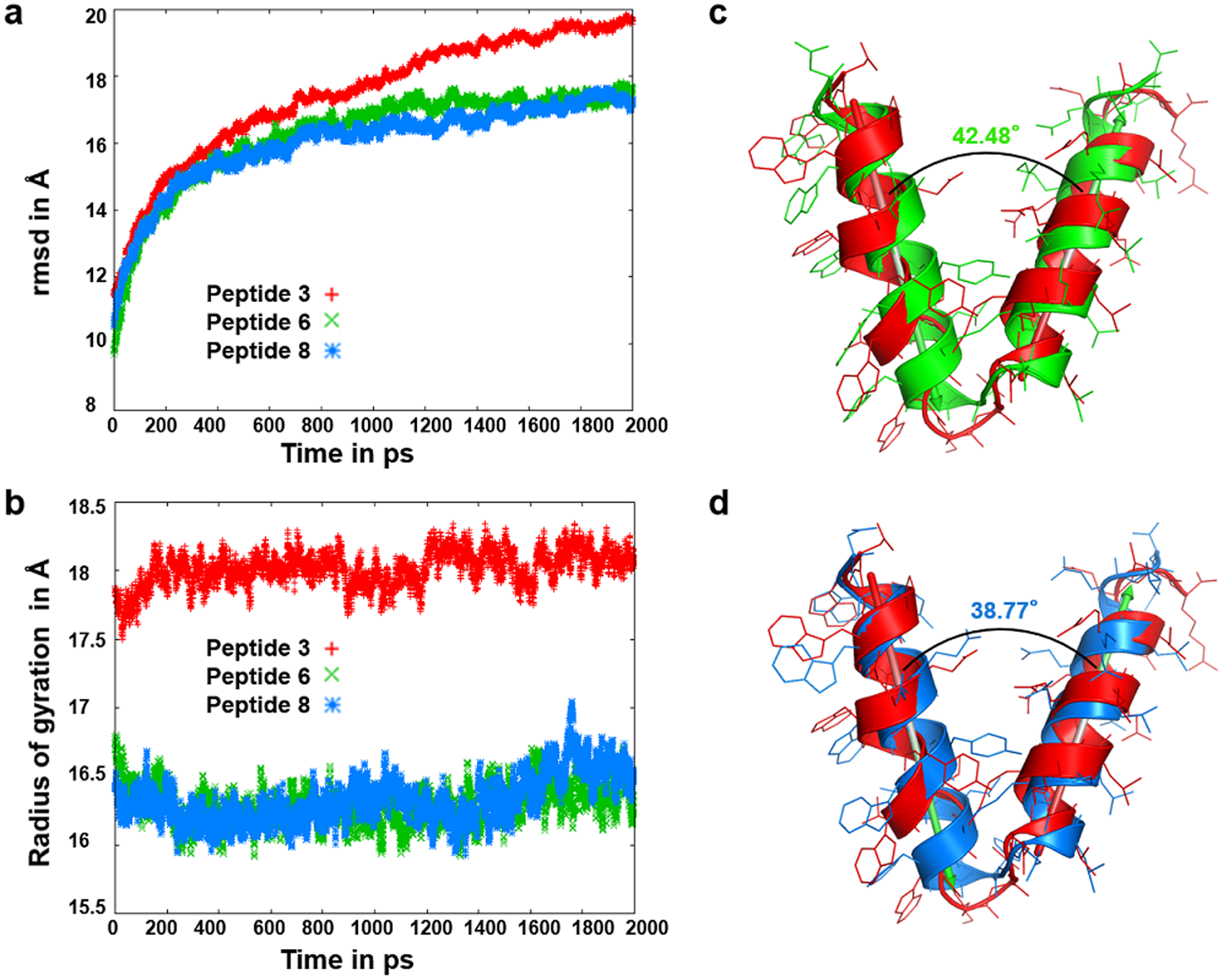

Supplement: Supplementary file 4 — Additional file 4. Figure S4. Molecular dynamics simulation of three peptides 3, 6, and 8 for 2 ns. RMS deviations (a) and radius of gyrations (b) of the three peptides 3, 6, and 8 relative to their corresponding energy minimized structures after 2 ns of MD simulation. (c) Superposition of the molecular structure of peptide 6 on peptide 3 after 2 ns of MD simulation. (d) Superposition of the molecular structure of peptide 8 on peptide 3 after 2 ns of MD simulation. [file 12951_2015_119_MOESM4_ESM.tif]
